# Does retinopathy predict stroke recurrence in type 2 diabetes patients: A retrospective study?

**DOI:** 10.1371/journal.pone.0210832

**Published:** 2019-01-17

**Authors:** Ola Hjelmgren, Ulf Strömberg, Karl Gellerman, Anders Thurin, Madeleine Zetterberg, Göran Bergström

**Affiliations:** 1 The Wallenberg Laboratory, Department of Molecular and Clinical Medicine, Institute of Medicine, Sahlgrenska Academy at the University of Gothenburg, Gothenburg, Sweden; 2 Department of Clinical Physiology, Sahlgrenska University Hospital, Gothenburg, Sweden; 3 Health Metrics Unit, Institute of Medicine, Sahlgrenska Academy, University of Gothenburg, Gothenburg, Sweden; 4 Department of Clinical Neuroscience and Rehabilitation/Ophthalmology, Institute of Neuroscience and Physiology, Sahlgrenska Academy at the University of Gothenburg, Gothenburg, Sweden; Weill Cornell Medicine-Qatar, QATAR

## Abstract

**Aims:**

To study if retinopathy increases the risk of stroke recurrence in stroke patients with type 2 diabetes. Also, to study if stroke patients with type 2 diabetes have an increased risk of stroke recurrence compared to non-diabetics and if stroke patients with type 2 diabetes, regardless of retinopathy, have a higher incidence of carotid stenosis. Also, to study if stroke patients with type 2 diabetes retinopathy have increased incidence of carotid stenosis.

**Methods:**

We included 445 patients with type 2 diabetes mellitus and a matched control group of 445 patients without diabetes, who had all suffered their first stroke or TIA. Information on retinopathy, risk factors and stroke recurrence were obtained from registers and medical records.

**Results:**

Retinopathy did not increase the risk of stroke recurrence in diabetes patients, HR 0.89 (0.51–1.53), p = 0.67. The risk of stroke recurrence was not increased in diabetics compared to non-diabetes. Diabetes patients had an increased prevalence of carotid stenosis compared to non-diabetics, 1.69 (1.15–2.48), p = 0.008. The prevalence of carotid stenosis in diabetics with retinopathy was not increased compared to diabetics without retinopathy.

**Conclusion:**

Retinopathy is not a predictor of stroke recurrence or carotid stenosis in type 2 diabetes patients.

## Introduction

Patients with type 2 diabetes mellitus (T2DM) have an increased risk of ischemic stroke and other cardiovascular diseases [1]. Furthermore, patients with diabetic retinopathy (DR), a common microvascular diabetes complication affecting the retinal vessels of the eye [2], have an even higher risk of ischemic stroke (IS) [3–9], as well as higher overall cardiovascular and all-cause mortality [3, 10, 11]. Embolic stroke from carotid artery stenosis (CAS) constitutes an important etiological subtype of ischemic stroke [12], and carotid surgery of symptomatic CAS reduces the risk of recurrent stroke [13]. The risk of recurrent stroke in patients with diabetes and CAS appears to be even further increased [14]. Interestingly, it has been suggested that the microvascular pathologies found in the retina of patients with DR may also be present in atherosclerotic plaque [15]. In fact, a recent study found signs of increased carotid vasa vasorum vascularization in patients with retinopathy [16]. This is of pathophysiological interest since the vascularization of atherosclerotic plaques has been suggested to both increase the growth rate of plaques and result in a more vulnerable plaque phenotype, suggesting a higher risk of stroke [17].

Whether the burden of carotid atherosclerosis is increased in patients with retinopathy is not clear. Some studies [18–22] have found a relationship between carotid plaque or stenosis and DR in asymptomatic populations, but the reports have not been unequivocal and never focused on an exclusively symptomatic population [16, 19, 20, 23–25]. Whether patients with DR and CAS are at increased risk of recurrent ischemic stroke compared with those without retinopathy has not been studied to our knowledge.

The primary aim of this retrospective register study was: To determine if DR increases the risk of stroke recurrence in stroke patients with T2DM. The secondary aims were: 1) To study if stroke patients with T2DM (regardless of retinopathy status) has an increased risk of stroke recurrence compared to non-diabetic stroke patients. 2) To study if stroke patients with T2DM (regardless of retinopathy status) have a higher incidence of CAS. 3) To study if stroke patients with DR have increased incidence of CAS compared to stroke patients with T2DM without retinopathy.

## Materials and methods

### Study population

The subjects in this retrospective cohort study were recruited from the Western Region Initiative to Gather Information on Atherosclerosis (WINGA) database. This database includes patients at Sahlgrenska University Hospital undergoing ultrasound examination for suspected cerebrovascular disease. The hospital is the sole supplier of vascular diagnostics in the greater Gothenburg region, with approximately 950,000 inhabitants. According to local guidelines, ultrasound of the carotid arteries is the recommended first-line diagnostics for carotid arteries in patients with suspected stroke or TIA. The study is thus representative for the absolute majority of patients in this region suffering from stroke or TIA and being referred for carotid ultrasound. We consecutively included patients who had suffered their first IS or TIA and been referred for carotid ultrasound examination between January 1^st^, 2004 and December 31^st^, 2010.

Inclusion criteria were: IS or TIA within 6 months before the ultrasound examination, age of 40 years or over and T2DM. All patients fulfilling the inclusion criteria were consecutively identified. The control group was a similar-sized group of patients, randomized from the same cohort but with no diabetes and matched for age, sex, type of presenting event and residential area. The study was approved by the local Regional Ethics Committee.

### Ascertainment of clinical data

Incident IS and TIA (IS ICD-10: I63, I64 and TIA ICD-10: G45and H34) within 6 months before the ultrasound examination were identified by matching patients in the WINGA registry with data from the Swedish National Inpatient Register (NIPR). To ascertain that the present event was the patient’s first ever cerebral event a search was made back to 1987 for any previous diagnosis of a cerebral event. The primary event was classified as ischemic stroke or TIA, and the TIA class also includes amaurosis fugax. Information on diabetes was collected and cross-validated from several sources: the Swedish National Diabetes Register (NDR), the Swedish National Drug Prescription Register (NDPR) and the NIPR. Patients were regarded as having diabetes mellitus (DM) if they had any diagnosis of DM in NDR or NIPR and/or were prescribed blood glucose-lowering medication at the year of IS/TIA or earlier.

We obtained medical records from all three eye clinics managing the screening for retinopathy amongst patients with T2DM in Gothenburg. DR was determined as either absent or present, the latter including all stages of DR, according to two strict and pre-specified criteria: i) DR was considered present if the patient had signs of DR in any fundus photograph taken before or less than six months after the ultrasound examination: ii) DR was defined as absent if no signs of DR were found after or within two years prior to the ultrasound examination.

Comorbidity at the time of the primary event was defined as the following diagnosis in NIPR: Coronary heart disease as a composite of myocardial infarction (ICD-10: I21), cardiac arrest (I46.0, I46.9), angina (I20.9), other ischemic heart disease (I20.0, I20.8, I20.9, I25.1, I25.9), or coronary intervention (Coronary Artery Bypass Grafting, CABG or Percutaneous Coronary Intervention, PCI). Peripheral artery disease (PAD) as a composite of claudication (I73.9) or critical limb ischemia (I70.2). Mechanical heart valve was defined as Z95.2. Atrial fibrillation was defined as ICD I48.

### Serum markers and smoking

Data on creatinine were extracted from digital patient records if analyzed within 30 days before and no longer than 2 days after the primary event. Hypertension was defined as systolic blood pressure > 140 mmHg or diastolic blood pressure > 90 mmHg. Smoking status was obtained from a text-based search of patient records from the year of event and defined as smoker/ex-smoker/non-smoker.

### Definition of carotid artery stenosis using ultrasound

The ultrasound (US) examinations were performed using an Acuson Sequoia 512 (Siemens Acuson, Garnerville, NY, USA). Carotid artery stenosis was defined as >50% carotid artery diameter obstruction due to atherosclerosis on either or both sides according to the European Carotid Surgery Trial criteria (ECST) [26]. If bilateral CAS was present, the grade of stenosis was determined according to the status in the most-affected side.

### Follow-up and outcome assessment

Patients were followed up for recurrent stroke events from the day of their inclusion in the study (date of ultrasound examination) until December 31, 2010. The NIPR and the National Death Register was searched to assess which patients had suffered a non-fatal or fatal event during the follow-up period. The primary endpoint was recurrent stroke, defined as any diagnosis of fatal or non-fatal ischemic stroke after the primary event and adjudicated by an evaluation of medical records and/or death certificates (ICD-10: I63, I64).

### Statistical analysis

Continuous variables are presented as means (standard deviation) or medians (quartiles). Categorical variables are presented as counts (%). T-tests or the Mann-Whitney U test were used in univariate analyzes of continuous variables. Categorical variables were analyzed using the chi^2^-test. For the presence of carotid stenosis and recurrence of stroke, logistic binary regression and Cox regression were used respectively to adjust for confounding factors. Assumption of proportionality in the survival analysis was not violated when analyzed visually. Patients who suffered from non-stroke death were censored in the Cox regression. Results from these analyses are presented as odds ratios (OR) and hazard ratios (HR) with 95% confidence intervals. Two-sided p-values <0.05 were considered statistically significant. IBM SPSS Statistics 24.0 (IBM, Armonk, NY) was used for all analyses.

## Results

### Study population

Between 2004 and 2010, 13,380 unique patients underwent carotid ultrasound at our clinic. Among those patients, 6,858 met the inclusion criteria of this study. Among these patients, we identified 1,333 patients as fulfilling the criteria for DM during the study period. Patients were subsequently excluded from the DM group if they did not have an established diagnosis of DM at or before the year of the primary event or where information about year of diabetes onset was missing (n = 665). Patients were excluded if a diabetes diagnosis other than type 2 was present (n = 47) or where information on diabetic retinopathy was absent or ambiguous (n = 176). Patients lacking information on retinopathy were older than those with information on retinopathy (mean 71.7 SD 9.4 vs. mean 68.9 SD 9.6 years, p = 0.001) while no other significant differences were observed regarding sex (male sex 60% vs. 65%, p = 0.30), Socio economic status (SES) (high 19% vs. 25%, middle 35% vs. 32% low 46% vs. 43%, p = 0.21), type of first event (Ischemic stroke 73% vs. 67%, p = 0.20), presence of carotid stenosis (73% vs. 77%, p = 0.31), duration of diabetes (mean 8.3 SD 7.5 vs. 7.9 7.0 years, p = 0.59) or stroke recurrence (12% vs. 14%, p = 0.81). Our study sample finally consisted of 445 patients with type 2 diabetes and information on DR, 204 of whom (46%) had retinopathy. A similar sized group of patients (n = 445) without DM and matched for age, sex, presenting event (stroke or TIA) and residential area were selected from the reminder of the cohort. Characteristics on all of the patients (DM patients and controls) are reported in Table 1. Characteristics on the DM patients (with and without retinopathy) are reported in Table 2. Residential area was used as a proxy for SES, classified as high, middle or low. The variable “stroke as type of first event” is the fraction of the subjects who debuted with an ischemic stroke in contrast to subjects who debuted with a TIA or amaurosis fugax.

**Table 1:** Comparison of clinical characteristics between diabetes patients and controls.

		Diabetes patients n = 445	Controls n = 445	p-value
Age, years		68.9 (9.6)	68.9 (9.6)	0.96
Male sex		288(64.7%)	288(64.7%)	1.0
Stroke as type of first event		145 (33%)	145 (33%)	1.0
Days from event to ultrasound		3 (1–5)	3 (1–6)	0.18
Carotid artery stenosis				
	<50%	341 (47.6%)	376 (52.4%)	0.008
	50–69%	42 (56%)	33 (44%)	
	≥70%	62 (63.3%)	36 (36.7%)	
Coronary Heart Disease		133 (30%)	76 (17%)	<0.001
Heart failure		49 (11%)	25 (6%)	<0.005
Peripheral Artery Disease		24 (5%)	13 (3%)	0.07
Atrial Fibrillation		63 (14%)	59 (13%)	0.70
Smoking				
	Non-smoker	127 (37%)	148 (42%)	0.26
	Ex-smoker	83 (24%)	85 (24%)	
	Current smoker	137 (40%)	120 (34%)	
Socioeconomic status				
	High	113 (25%)	113 (25%)	1.0
	Middle	142 (32%)	142 (32%)	
	Low	190 (43%)	190 (43%)	
Hypertension			293(70%) (n = 417)	262(64%) (n = 407)	0.07
Creatinine, μmol/l		77 (63–98) n = 396	80 (69–95) n = 384	0.10
Mechanical heart valve		3 (0.7%)	3 (0.7%)	1.0

**Table 2:** Comparison of clinical characteristics between diabetes patients with and without retinopathy.

		No Retinopathy n = 241	Retinopathy n = 204	p
Age, years		68.8 (9.6)	69.0 (9.7)	0.81
Male sex		153(64%)	135 (66%)	0.62
Stroke as type of first event		158 (66%)	142 (70%)	0.42
Days from event to ultrasound		3 (1–5)	2 (1–4)	0.26
Carotid artery stenosis				
	<50%	185 (77%)	156 (76%)	0.15
	50–69%	19 (8%)	23 (11%)	
	≥70%	37 (15%)	25 (12%)	
Coronary Heart Disease		73 (30%)	60 (29%)	0.84
Heart failure		29 (12%)	20 (10%)	0.45
Peripheral Artery Disease		13 (5%)	11 (5%)	1.0
Atrial Fibrillation		35 (15%)	28 (14%)	0.81
Duration of diabetes (years)		6.1 (5.4)	10.2 (8.0)	<0.001
Smoking				
	Never smoked	69 (37%)	58 (36%)	
	Ex-smoker	46 (25%)	37 (23%)	0.91
	Current smoker	72 (39%)	65 (41%)	
Socioeconomic status				
	High	61 (25%)	52 (26%)	0.91
	Middle	75 (31%)	67 (33%)	
	Low	105 (44%)	85 (42%)	
Hypertension		150 (70%) (n = 224)	143 (74%) (n = 193)	0.11
Creatinine, μmol/l		84.0 (46.6) (n = 214)	88.4 (35.1) (n = 182)	0.12
Mechanical heart valve		1 (0.4%)	2 (1%)	0.47

### Stroke recurrence in diabetes patients with and without retinopathy

In the diabetes group, there were 34 events of recurrent stroke in the retinopathy group (14.1%) and 28 events of recurrent stroke in the non-retinopathy group (13.7%). The bivariate HR for recurrent stroke in diabetics with retinopathy was 0.99 (0.60–1.63), p = 0.96. Variables with bivariate HR with p <0.20 were entered in the adjusted model. The bivariate HR for recurrent stroke in patients with dyslipidemia was 1.63 (0.86–3.09), p = 0.14. Dyslipidemia was excluded from the adjusted model since almost 50% of the patients with recurrent stroke lacked data on dyslipidemia.

After adjustment for age, sex, duration of diabetes and type of primary event, there was no increased risk for recurrent stroke in the retinopathy group, adjusted HR 0.89 (0.51–1.53), p = 0.67 (Table 3).

**Table 3:** Cox proportional hazards model in diabetes patients with recurrent stroke as endpoint.

		Bivariate HR (95% CI	p	Model I	p
Age			0.03		0.07
	<61 years	1 (ref)		1 (ref)	
	61–65	0.79 (0.32–1.94)	0.61	0.85 (0.34–2.11)	0.72
	66–70	0.27 (0.08–0.96)	0.04	0.26 (0.07–0.93)	0.04
	71–75	1.26 (0.57–2.75)	0.57	1.18 (0.53–2.61)	0.69
	76–80	1.95 (0.93–4.05)	0.08	1.76 (0.83–3.71)	0.14
	>80	1.46 (0.59–3.57)	0.41	1.20 (0.47–3.07)	0.71
Male sex		0.64 (0.39–1.06)	0.08	0.73 (0.44–1.23)	0.24
**Diabetes with retinopathy**		**0.99 (0.60–1.63)**	**0.96**	**0.89 (0.51–1.53)**	**0.67**
Duration of diabetes, quartiles			0.15		0.31
	<2 years	1 (ref)		1 (ref)	
	2–6	1.70 (0.73–3.93)	0.22	1.78 (0.77–4.13)	0.18
	7–12	2.21 (0.97–5.06)	0.06	1.99 (0.86–4.62)	0.11
	>12	2.51 (1.09–5.79)	0.03	2.30 (0.94–5.63)	0.07
Stroke as type of first event		1.6 (0.88–2.90)	0.12	1.63 (0.89–2.96)	0.11
SES group			0.60		
	High	1 (ref)			
	Middle	1.28 (0.63–2.59)	0.5		
	Low	1.4 (0.73–2.68)	0.31		
Carotid artery stenosis			0.25		
	<50%	1(ref)			
	50%–69%	1.14 (0.49–2.69)	0.76		
	70%–89%	1.21 (0.38–3.91)	0.75		
	> = 90%	1.99 (1.02–3.89)	0.04		
Coronary heart disease		1.40(0.83–2.36)	0.21		
Heart failure		1.13(0.51–2.47)	0.77		
Peripheral artery disease		1.35(0.49–3.71)	0.57		
Atrial fibrillation		0.78 (0.35–1.71)	0.53		
Smoking			0.78		
	Not smoker	1 (ref)			
	Ex-smoker	0.81(0.39–1.67)	0.56		
	Smoker	0.82(0.45–1.52)	0.54		
Creatinine, quartiles			0.25		
	<64	1 (ref)			
	64–77	1.03 (0.52–2.04)	0.93		
	78–98	0.47 (0.20–1.10)	0.08		
	>98	1.04 (0.52–2.08)	0.91		
Hypertension		0.95 (0.54–1.67)	0.86		
Mechanical heart valve		0.05 (0–39197)	0.66		

**Model I:** All variables with bivariate HR with p <0.20 were entered in the adjusted model

### Stroke recurrence in diabetes and non-diabetes patients

**Fig 1:**
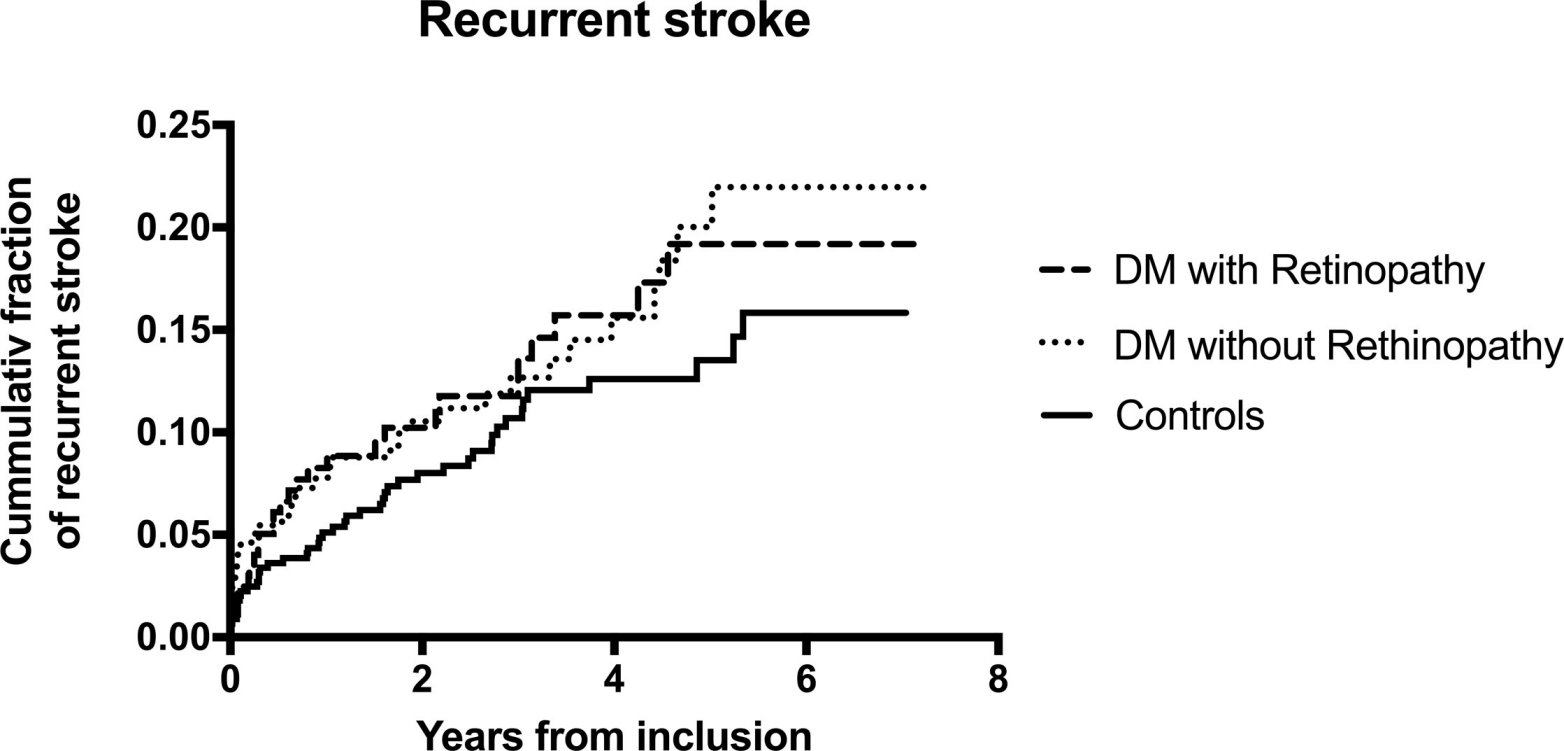
Kaplan-Meier curve of the cumulative proportion with recurrent stroke. P-value for the difference between patients with and without diabetes was 0.07. P-value for the difference between diabetes patients with and without retinopathy was 0.96.

Recurrent stroke occurred in 108 of 890 (12%) of the patients (DM and controls), and the median follow up time was 3.1 years. In the diabetes group, 62 patients (14%) suffered from recurrent stroke compared with 46 patients (10%) in the control group (bivariate HR 1.42, 95% (0.97–2.07), p = 0.07) (Fig 1). Factors associated with recurrence were age, having stroke as the primary event, carotid stenosis, coronary heart disease and heart failure. Controls were matched for age, sex, socioeconomic status and type of primary event. Variables with bivariate HR with p <0.20 were entered in the adjusted model. After adjustment for age, sex, presence of diabetes, type of primary event, degree of carotid stenosis and heart failure, the adjusted hazard ratio for diabetes was 1.30 (0.88–1.91), p = 0.20 (Table 4).

**Table 4:** Cox proportional hazards model in all subjects with recurrent stroke as endpoint.

		Bivariate HR	p	Model I	p
Age			<0.01		<0.01
	<61 years	1 (ref)		1 (ref)	
	61–65	0.87 (0.44–1.72)	0.68	0.78(0.39–1.56)	0.49
	66–70	0.51(0.23–1.13)	0.10	0.45(0.20–1.00)	0.05
	71–75	1.35(0.74–2.48)	0.33	1.21(0.65–2.25)	0.54
	76–80	1.88(1.07–3.32)	0.03	1.82(1.03–3.24)	0.04
	>80	1.49(0.74–3.00)	0.27	1.37(0.67–2.78)	0.39
Male sex		0.73(0.50–1.07)	0.11	0.76(0.51–1.13)	0.18
**Presence of Diabetes**		**1.42(0.97–2.07)**	**0.07**	**1.30 (0.88**–**1.91)**	**0.20**
SES group			0.57		
	High	1 (ref)			
	Middle	1.32(0.79–2.21)	0.30		
	Low	1.21(0.74–1.98)	0.45		
Stroke as type of first event		1.82(1.14–2.91)	0.01	1.89 (1.18–3.03)	<0.01
Carotid artery stenosis			0.04		0.03
	<50%	1 (ref)		1 (ref)	
	50–69%	1.25 (0.65–2.42)	0.50	1.12(0.57–2.20)	0.70
	70–89%	1.11 (0.41–3.02)	0.84	1.16(0.42–3.21)	0.78
	> = 90%	2.26 (1.30–3.93)	<0.01	2.42(1.37–4.27)	<0.01
Coronary heart disease		1.56 (1.04–2.35)	0.03	1.27(0.81–2.01)	0.30
Heart failure		1.52(0.83–2.77)	0.17	0.67(0.60–2.23)	0.67
Periphery artery disease		1.63 (0.72–3.73)	0.24		
Atrial fibrillation		0.98 (0.56–1.72)	0.95		
Smoking			0.66		
	Not smoker	1 (ref)			
	Ex-smoker	0.82 (0.47–1.42)	0.48		
	Smoker	1.06 (0.67–1.65)	0.82		
Creatinine, quartiles			0.50		
	<66	1 (ref)			
	66–78	0.70(0.41–1.22)	0.21		
	79–96	0.69(0.41–1.19)	0.18		
	>96	0.80(0.48–1.35)	0.41		
Hypertension		0.96 (0.64–1.44)	0.85		
Mechanical heart valve		0.05 (0–616)	0.53		

**Model I:** Variables with bivariate HR with p <0.20 were entered in the adjusted model.

### Incidence of carotid stenosis in diabetes and non-diabetes patients

Carotid stenosis was present in 173 of 890 (19%) patients. The number of patients with carotid stenosis in the diabetes population was 104 (23%), compared with 69 (16%) in the controls, bivariate OR 1.66 95% (1.19–2.33), p = 0.003). Factors associated with carotid artery stenosis were age, coronary heart disease, heart failure and periphery artery disease. Controls were matched for age, sex, socioeconomic status and type of primary event. Variables with bivariate HR with p <0.20 were entered into the adjusted model. After adjustment for age, SES, coronary heart disease, heart failure, periphery artery disease and creatinine, patients with diabetes had an increased risk for having carotid artery stenosis compared to controls, OR 1.69 (1.15–2.48), p = 0.008 (Table 5).

**Table 5:** Logistic regression in all subjects with presence of carotid stenosis as outcome.

		Bivariate OR	p	OR Model I	p
Age			<0.01		0.02
	<61 years	1 (ref)		1 (ref)	
	61–65	1.98 (1.04–3.78)	.04	1.83(0.90–3.74)	0.10
	66–70	3.15 (1.72–5.76)	<0.001	3.12(1.62–6.02)	<0.01
	71–75	2.87 (1.57–5.27)	.001	2.22(1.13–4.38)	0.02
	76–80	1.85 (0.98–3.48)	.06	1.53(0.75–3.16)	0.25
	>80	2.54 (1.28–5.06)	<0.01	2.50(1.18–5.33)	0.02
Male sex		1.17 (0.83–1.67)	0.37		
**Presence of Diabetes**		**1.66 (1.19–2.33)**	**0.003**	**1.69(1.15–2.48)**	**0.008**
SES group			0.19		0.05
	High	1 (ref)		1 (ref)	
	Middle	1.34(0.84–2.13)	0.22	1.20(0.7–2.03)	0.51
	Low	1.50 (0.97–2.32)	0.07	1.76(1.08–2.88)	0.02
Stroke as type of first event		0.92 (0.65–1.31)	0.64		
Coronary heart disease		2.17 (1.51–3.10)	<0.001	1.27(0.81–2.00)	0.30
Heart failure		2.15 (1.28–3.61)	<0.01	1.58(0.86–2.92)	0.14
Peripheral artery disease		3.80 (1.94–7.42)	<0.001	2.94(1.33–6.47)	0.08
Atrial fibrillation		1.28 (0.81–2.03)	0.29		
Smoking			0.35		
	Not smoker	1 (ref)			
	Ex-smoker	1.42(0.88–2.29)	0.15		
	Smoker	1.23(0.8–1–91)	0.35		
Creatinine, quartiles			0.17		0.17
	<66	1 (ref)			
	66–78	1.43(0.83–2.46)	0.20	1.67(0.95–2.95)	0.08
	79–96	1.65(0.97–2.80)	0.07	1.86(1.06–3.28)	0.03
	>96	1.77(1.05–3.01)	0.03	1.58(0.86–2.92)	0.14
Hypertension		1.00 (0.69–1.44)	0.99		
Mechanical heart valve		0.83 (0.10–7.13)	0.86		

**Model I:** All variables with p <0.2 were entered in the model.

### Incidence of carotid stenosis in diabetes patients with and without retinopathy

Carotid stenosis was equally common in diabetes patients with (n = 48, 24%) and without (n = 56, 23%) retinopathy, bivariate OR 1.02 (0.65–1.58), p = 0.94. Factors associated with carotid artery stenosis were age, coronary heart disease, heart failure and periphery artery disease. Variables with bivariate HR with p <0.20 were entered in the adjusted model. After adjustment for age, coronary heart disease, heart failure, periphery artery disease and creatinine, patients with retinopathy had no increased risk of having carotid artery stenosis, OR 0.79 (0.48–1.30), p = 0.35 (Table 6).

**Table 6:** Logistic regression in diabetes subjects with presence of carotid stenosis as outcome.

		Bivariate OR	p	Model I	p
Age			0.06		0.08
	<61 years	1 (ref)		1 (ref)	
	61–65	2.57 (1.16–5.69)	0.02	2.22 (0.93–5.32)	0.07
	66–70	2.61 (1.20–5.70)	0.02	2.66 (1.16–6.11)	0.02
	71–75	2.48 (1.14–5.39)	0.02	2.06 (0.88–4.81)	0.10
	76–80	1.21 (0.52–2.84)	0.66	0.94 (0.36–2.43)	0.90
	>80	2.14 (0.88–5.24)	0.10	2.03 (0.76–5.38)	0.16
Male sex		1.04 (0.67–1.65)	0.87		
**Presence of retinopathy**		**1.02 (0.65–1.58)**	**0.94**	**0.79 (0.48–1.30)**	**0.35**
Duration of diabetes. quartiles			**0.63**		
	<2 years	1 (ref)			
	2–6	1.07 (0.55–2.06)	0.85		
	7–12	1.44 (0.75–2.74)	0.27		
	>12	1.32 (0.67–2.57)	0.42		
SES group			0.38		
	High	1 (ref)			
	Middle	1.49 (0.81–2.73)	0.20		
	Low	1.44 (0.81–2.57)	0.22		
Stroke as type of first event		1.05 (0.66–1.68)	0.83		
Coronary heart disease		1.76 (1.11–2.79)	0.02	1.16 (0.65–2.07)	0.63
Heart failure		1.89 (1.00–3.56)	0.05	1.41 (0.66–3.00)	0.38
Peripheral artery disease		4.29 (1.86–9.89)	0.001	3.96 (1.48–10.58)	<0.01
Atrial fibrillation		0.93 (0.49–1.76)	0.82		
Smoking			0.65		
	Not smoker	1 (ref)			
	Ex-smoker	0.73 (0.38–1.43)	0.36		
	Smoker	0.94 (0.54–1.64)	0.83		
Creatinine, quartiles			0.19		0.38
	<64	1 (ref)		1 (ref)	
	64–77	1.18 (0.58–2.40)	0.64	1.11(0.71–2.97)	0.77
	78–98	1.51 (0.76–3.01)	0.24	1.41(0.71–2.97)	0.35
	>98	1.99 (1.02–3.89)	0.04	1.82(0.94–3.95)	0.11
Hypertension		0.79 (0.48–1.28)	0.33		
Mechanical heart valve		1.65 (0.15–18.3)	0.69		

**Model I:** All variables with bivariate HR with p <0.20 were entered into the adjusted model.

## Discussion

This is the first study to examine the relationship between DR, CAS and recurrent stroke in an exclusively symptomatic population. In this study, T2DM was associated with an increased prevalence of CAS, but the association with recurrent stroke was not significant. Furthermore, DR was not associated with CAS and not associated with recurrent stroke.

Atherosclerosis in the carotid artery is very common, but not all patients develop symptoms [27, 28]. Patients who develop symptoms from their plaque therefore must have special characteristics or have been exposed to certain stimuli compared to patients who remains asymptomatic [29]. Hallmarks of DR include increased vascular permeability, hemostatic abnormalities, endothelial dysfunction, increased tissue ischemia, and neoangiogenesis [30]. Presence of DR may be a marker of a more generalized micro-vessel dysfunction present also in the plaque [15]. Micro-vessels have been linked to plaque growth and vulnerability [17] as well as future cardiovascular events [31]. Thus, it is conceivable that diabetes patients with DR who suffered an ischemic stroke may be at increased risk of recurrent ischemic stroke compared with those without DR.

Interestingly, while the increased risk of stroke in previously asymptomatic patients with diabetic DR has been firmly established [4–9], we found no such association in our study of symptomatic patients with and without CAS. It must be acknowledged that we did not have detailed information on the exact location or subtype of the strokes in our study, which could have reduced our ability to detect differences. Nevertheless, the differences between patients with and without DR with regard to stroke recurrence were small, indicating that DR is not a strong predictor of increased risk of stroke recurrence.

Our subgroup analysis of patients with diabetes and CAS revealed no associations between DR and outcome. While the number of patients in this analysis was limited, making it difficult to attain significant associations, the absolute difference between patients with and without DR was small. Previous studies, investigating stroke incidence in mostly asymptomatic patients with DR, unfortunately did not examine the presence of CAS [4–9]. Thus, it is not known whether DR indicates an increased risk of stroke also in asymptomatic patients with CAS. It is possible that DR affects early pathological vascularization of plaque tissue and thus reflects an increased risk of asymptomatic plaques turning into symptomatic ones. However, the current cohort was made up of symptomatic patients, and it may be that we have a more advanced plaque phenotype and that DR is no longer a strong predictor of plaque vulnerability.

A relationship between carotid plaque size and DR has been found in some studies[20–22], while no such association was seen in others [16, 19, 23, 25, 32, 33]. Arcidiacono et al. [16] found an increased contrast agent uptake in the sub-intimal space of asymptomatic patients with DR, suggesting increased vasa vasorum density. Also, they found a potential trend towards larger carotid intima media thickness and plaque presence in the DR group [16]. However, after adjusting for other cardiovascular risk factors, these associations were diminished. In studies which included patients with previous stroke, the absolute numbers of such patients were either small [19, 20, 23] or not stated [21, 25], and no separate analysis was made for this subgroup. Furthermore, the timing between symptom and ultrasound examination was not clear. We now extend these observations by showing a lack of significant association between presence of CAS and DR in a large group of patients with recent symptoms of cerebral ischemia. Based on the inconsistent results of previous studies as well as our current data, we may conclude that the effect of DR on the extent of carotid atherosclerosis is not strong in either symptomatic or asymptomatic patients.

### Limitations

The population size is limited, although it compares reasonably well with previous study cohorts focusing on CAS. We did not have detailed information on carotid atherosclerosis, and the analysis is cross-sectional and performed in a rather late and advanced phase of the disease, thus, it does not exclude that DR may affect plaque size if a more detailed analyses were to be done or if earlier time points in plaque development were chosen. In addition, due to the limited population size, no subgroup analysis of patients with different severity of DR was done, *i*.*e*. we do not know whether patients with advanced stages of DR such as neovascularization or vitreous hemorrhage differ in their risk of recurrent stroke compared to patients with mild-to-moderate DR. In this study, we have no data on serum lipid levels or medication of the patients. Missing data on some potentially confounding variables is a weakness, although the bivariate HR for recurrent stroke in diabetics with DR was 0.99, p = 0.96. It is therefore unlikely that adjustment for these potential confounders would significantly alter the HR.

Patients who had undergone ultrasound examination were included in this study, which may have led to selection bias since the most severely ill patients are most likely not referred to ultrasound examinations since they may be deemed unsuitable for carotid artery surgery. This is a hospital-based cohort, and any extrapolation of our results to the general population should be done with caution.

## Conclusion

In summary, our study of T2DM patients with their first ischemic stroke or TIA found no association between DR and recurrent stroke and no significant association between diabetes and recurrent stroke. We found an association between the presence of CAS and diabetes but no association between DR and the presence of CAS. We conclude that DR, in this study, is not an indicator of the presence of carotid artery stenosis or the risk of recurrent stroke.
